# Genistein Disrupts Glucocorticoid Receptor Signaling in Human Uterine Endometrial Ishikawa Cells

**DOI:** 10.1289/ehp.1408437

**Published:** 2014-08-19

**Authors:** Shannon Whirledge, Linda T. Senbanjo, John A. Cidlowski

**Affiliations:** Laboratory of Signal Transduction, National Institute of Environmental Health Sciences, National Institutes of Health, Department of Health and Human Services, Research Triangle Park, North Carolina, USA

## Abstract

Background: The link between environmental estrogen exposure and defects in the female reproductive tract is well established. The phytoestrogen genistein is able to modulate uterine estrogen receptor (ER) activity, and dietary exposure is associated with uterine pathologies. Regulation of stress and immune functions by the glucocorticoid receptor (GR) is also an integral part of maintaining reproductive tract function; disruption of GR signaling by genistein may also have a role in the adverse effects of genistein.

Objective: We evaluated the transcriptional response to genistein in Ishikawa cells and investigated the effects of genistein on GR-mediated target genes.

Methods: We used Ishikawa cells as a model system to identify novel targets of genistein and the synthetic glucocorticoid dexamethasone through whole genome microarray analysis. Common gene targets were defined and response patterns verified by quantitative real-time reverse-transcription polymerase chain reaction. The mechanism of transcriptional antagonism was determined for select genes.

Results: Genistein regulated numerous genes in Ishikawa cells independently of estradiol, and the response to coadministration of genistein and dexamethasone was unique compared with the response to either estradiol or dexamethasone alone. Furthermore, genistein altered glucocorticoid regulation of GR target genes. In a select set of genes, co-regulation by dexamethasone and genistein was found to require both GR and ERα signaling, respectively.

Conclusions: Using Ishikawa cells, we observed that exposure to genistein resulted in distinct changes in gene expression and unique differences in the GR transcriptome.

Citation: Whirledge S, Senbanjo LT, Cidlowski JA. 2015. Genistein disrupts glucocorticoid receptor signaling in human uterine endometrial Ishikawa cells. Environ Health Perspect 123:80–87; http://dx.doi.org/10.1289/ehp.1408437

## Introduction

Environmental compounds with estrogenic activity, present in plant constituents, plastics, and pesticides, are recognized endocrine disruptors, leading to impaired reproductive function in a number of species (Caserta et al. 2008). Some of these compounds display a structure similar to that of the natural ligand and are able to physically interact with the estrogen receptors (ER), mimicking the activity of estradiol (E_2_) (Gray et al. 2002). However, unlike the biological effects of E_2_, which are regulated by feedback from the hypothalamic–pituitary–gonadal axis, dietary exposure to phytoestrogens is not under the control of feedback mechanisms and can potentially negatively affect reproductive tract function (Christensen et al. 2012). Due to the reported health benefits, consumption of soy in the United States has increased since the early 1990s (Adlercreutz et al. 1992; Beaglehole 1990). Soy is present as a food additive or meat substitute in up to 60% of processed foods, and soy formula is estimated to constitute around 12% of the infant formula market, a recent decrease from historically higher levels (Barrett 2006). Although soy is reported to have antioxidant and anticancer properties, the adverse effects of phytoestrogens on reproduction in animals are well established (Ravindranath et al. 2004). Infertility was initially described in 1946 in sheep foraging on red clover, an abundant source of phytoestrogens (Bennetts et al. 1946). Among the soybean isoflavones, genistein (Gen) is the most abundant and well characterized (Murphy et al. 2002). Infertility in captive cheetahs was attributed to the high Gen content in their diets and was reversed upon withdrawal of the soy-based diet (Setchell et al. 1987). These examples suggest that phytoestrogens exist in our environment at levels high enough to cause infertility in mammals, and that the pervasive use of phytoestrogens in food shows that humans and animals are unavoidably exposed to these compounds.

In addition to estrogenic activities, Gen can regulate the immune response (Masilamani et al. 2012). Gen has been reported to regulate human monocyte–derived dendritic cell maturation, secretion of dendritic cell–derived cytokines, and dendritic cell–mediated effector functions in culture (Wei et al. 2012). Interference of immune cell activation by Gen exposure may reflect one mechanism by which Gen causes infertility in mammals.

Classically, anti-inflammatory actions within the immune system are attributed to signaling by endogenous glucocorticoids and synthetic glucocorticoids given therapeutically (Busillo and Cidlowski 2013). Glucocorticoids mediate their biological functions through binding the glucocorticoid receptor (GR), a ligand-dependent transcription factor belonging to the nuclear receptor superfamily (Baxter and Tomkins 1970). Transcriptional antagonism through GR and ER binding to promoter elements in the glucocorticoid-induced leucine zipper (*GILZ*) gene was recently described in an immortalized human uterine endometrial cell line (Whirledge and Cidlowski 2013). Through binding ER, it is possible that Gen, like E_2_, antagonizes glucocorticoid-induced gene expression in this uterine cell model. Furthermore, overlap in the immune-modulatory functions ascribed to both Gen and glucocorticoids suggest that these hormones may target common cellular functions, and that exposure to Gen may alter the physiological role of glucocorticoids. In the present study, we used Ishikawa cells as a model to evaluate potential transcriptional antagonism of Gen and glucocorticoids. Special emphasis was given to co-regulated genes, particularly Gen-mediated changes to glucocorticoid-induced genes. Gen-mediated antagonism of the glucocorticoid-regulated transcriptome in Ishikawa cells may indicate one mechanism by which Gen exposure may alter the actions of glucocorticoids.

## Materials and Methods

*Reagents*. RPMI Medium 1640 (RPMI 1640; Gibco) was purchased from Invitrogen (Carlsbad, CA). Phenol Red-Free RPMI 1640 was prepared at the Media Unit of the National Institute of Environmental Health Sciences. We purchased heat-inactivated fetal bovine serum (FBS) from Atlanta Biologicals (Lawrenceville, GA); charcoal-stripped heat inactivated FBS from Hyclone (Logan, UT); dexamethasone (Dex), E_2_, and mifepristone (RU486) from Steraloids Inc. (Newport, RI); fulvestrant (ICI 182,780; ICI) and Gen from Sigma-Aldrich (St. Louis, MO); bisphenol A (BPA) from Midwest Research Institute (Kansas City, MO); and cycloheximide from Calbiochem-Novabiochem Corporation (La Jolla, CA). We obtained TaqMan® real-time reverse-transcription polymerase chain reaction (RT-PCR) primer probes from Applied Biosystems (Foster City, CA) and ON-TARGET*plus*® Control Pool (non-targeting pool) and SMARTpool (human *NR3C1*, *ER*α, and *ER*β) small-interfering RNA (siRNA) from Thermo Scientific (Waltham, MA).

*Culture of human Ishikawa cells*. We obtained an immortalized uterine human endometrial adenocarcinoma cell line (Ishikawa) from ATCC (American Type Culture Collection, Manassas, VA). Cells were grown in a standard tissue culture incubator at 37°C, with 95% humidity and 5% carbon dioxide. Ishikawa cells were maintained in RPMI 1640 supplemented with 5% FBS. Twenty-four hours prior to cell treatment, media were changed to phenol red–free RPMI 1640 containing 5% charcoal-stripped heat-inactivated FBS. Cells were treated with 100 nM Dex dissolved in phosphate-buffered saline (PBS) or with or without 10 nM E_2_, 100 nM Gen, or 100 nM BPA dissolved in ethyl alcohol (EtOH; Warner-Graham Company, Cockeysville, MD) for 6 hr. EtOH diluted in PBS to a final concentration of 0.1% served as the vehicle control for all studies. For GR or ER antagonism experiments, 1 μM RU486 or 1 μM ICI 182,780 prepared in EtOH was added 1 hr prior to agonist treatment. In cyclohexamide experiments, 10 μg/mL cyclohexamide was added 1 hr before agonist treatment.

*Quantitative RT-PCR (QRT-PCR)*. RNA isolation and QRT-PCR were performed as previously described (Whirledge et al. 2013). For additional information, see Supplemental Material, “RNA isolation and QRT-PCR.” The signal obtained from each gene primer–probe set was normalized to that of the unregulated housekeeping gene peptidylprolyl isomerase B (*PPIB*). Each gene’s primer–probe set was evaluated in at least four biological replicates of RNA.

*Microarray study*. We performed gene expression analysis using Agilent Whole Human Genome 4 × 44 multiplex format oligo arrays (Agilent Technologies, Santa Clara, CA) following the Agilent one-color microarray-based gene expression analysis protocol and has been previously described (Whirledge et al. 2013). For additional information see Supplemental Material, “Microarray study.” A heat map was generated using HeatPlus, Version 2.10 (BioConductor; http://www.bioconductor.org/packages/release/bioc/html/Heatplus.html). Normalized data and the averages of sample replicates of all significant probes were used for calculation of pairwise correlation. Dendrograms of samples and genes were generated by hierarchical clustering. The color scale ranged from 3-fold lower (log2-fold = –1.58) than mean to 3-fold higher (log2-fold = 1.58) than the mean. The lists of significant probe sets by treatment were visually sorted by Venn diagram (http://www.pangloss.com/seidel/Protocols/venn.cgi) and further analyzed by Ingenuity Pathway Analysis (IPA; version 6.5; Ingenuity Systems, Redwood City, CA). The average expression value of duplicate identifiers for the same molecule was used in the analyses to eliminate redundancy.

*Western blotting analysis*. We prepared whole cell lysates and performed Western blots as previously described (Whirledge and Cidlowski 2013). For additional information see Supplemental Material, “Whole cell lysates.” The protein was probed with polyclonal anti-GR antibody made by our laboratory (1:1,000), monoclonal anti-ER antibody (1:750; Immunotech, Marseille, France), polyclonal anti-GILZ antibody (1:500; Santa Cruz Biotechnology, Santa Cruz, CA), or monoclonal anti-β-actin antibody (1:10,000; Millipore, Temecula, CA).

*RNA interference*. For each biological replicate, cells were plated in six-well plates at approximately 70% confluence 1 day before transfection. Each siRNA (50 nM) was transfected into cells with DharmaFECT® 1 transfection reagent (Thermo Scientific) following the manufacturer’s instructions. The next day, each transfected well was divided into four wells of a six-well plate for RNA isolation after agonist treatment and one 10-cm dish for protein isolation. Forty-eight hours after transfection, the medium was changed to phenol red–free RPMI 1640. After 72 hr of siRNA treatment, cells were induced with 100 nM Dex, 100 nM Gen, or Dex + Gen; and mRNA was harvested 6 hr later.

*Chromatin immunoprecipitation assays*. Chromatin immunoprecipitation (ChIP) assays were performed using the Magna ChIP A Chromatin Immunoprecipitation Kit (Millipore, Billerica, MA) according to the manufacturer’s protocol as described previously (Whirledge and Cidlowski 2013). For additional information see Supplemental Material, “Chromatin immunoprecipitation assay.” Formalin-fixed chromatin was immunoprecipitated overnight with 10 μL of polyclonal GR antibody (Cidlowski et al. 1990), 10 μL of monoclonal ER antibody, or 2 μL of IgG (Millipore). PCR analysis of the GR response element (GRE), transcriptional start site (TSS), and control sequences utilized specific primers (see Supplemental Material, Table S1). The JASPAR database (http:jaspar.genereg.net) was used to identify predicted GR and ERα binding sites with 3,000 basepairs (bp) upstream and 500 bp downstream of the TSS. Sequences were scanned at a profile threshold score of 85%.

*Statistical analysis*. Data are presented as mean ± SE. Statistical significance was determined by analysis of variance with Tukey’s post hoc analysis. Statistical significance is reported as either *p* < 0.05 or *p* < 0.01.

## Results

**Figure 1:**
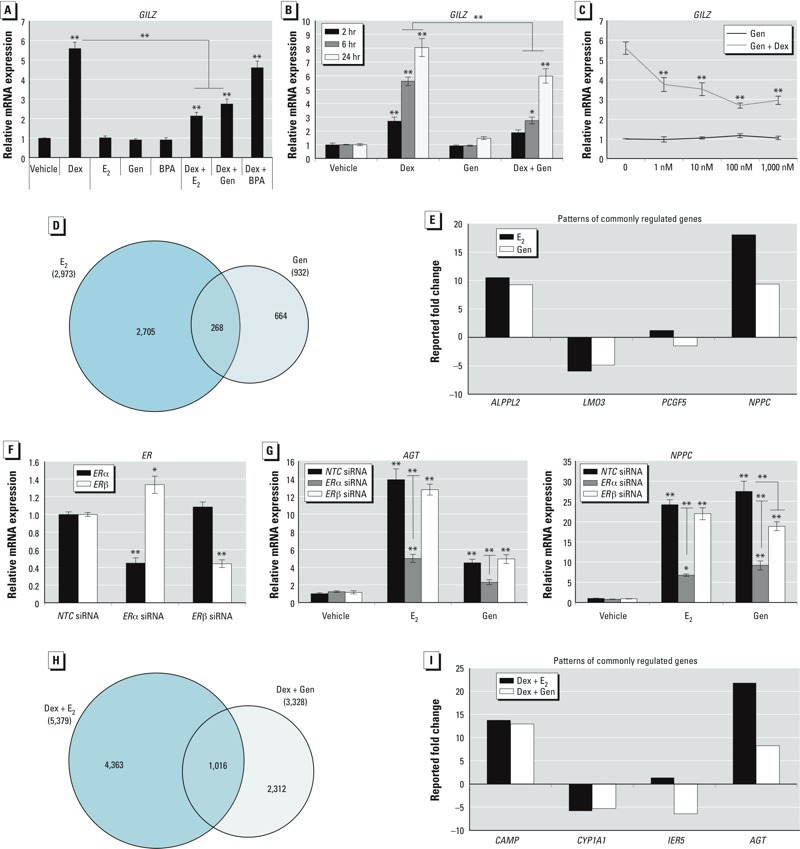
Gen regulated gene expression in Ishikawa cells independently of E_2_. (*A*) Expression of *GILZ* mRNA measured by QRT‑PCR following 6 hr treatment with vehicle (Veh), 100 nM Dex, 10 nM E_2_, 100 nM Gen, 100 nM BPA, or Dex + E_2,_ Dex + Gen, or Dex + BPA (concentrations used in combination were the same as independent treatment). (*B*) *GILZ* mRNA measured at 2, 6, and 24 hr after same treatment as in (*A*). (*C*) Expression of *GILZ* measured in cells treated for 6 hr with Veh, 0 nM–1,000 nM Gen, or 1 nM–1,000 nM Gen plus 100 nM Dex. (*D*) mRNA isolated from three biological replicates treated with E_2_ or Gen for 6 hr were analyzed by the Agilent Whole Human Genome 4 × 44 multiplex format oligo array for gene expression, and the number of probes statistically different (*p *< 0.01) between treatment groups were sorted by Venn diagram. (*E*) E_2_ and Gen co-regulated genes were separated by the direction of regulation; one gene representing each pattern of regulation is displayed (induced, repressed, anti-correlated, and antagonized, from left to right). (*F*) Cells transfected with non-targeting control pool (*NTC*), *ER*α, or *ER*β siRNA were assessed for the extent of ERα and ERβ knockdown by QRT‑PCR (*n* = 4). (*G*) *AGT* (angiotensinogen; left) and *NPPC* (natriuretic peptide type C; right) mRNA measured in transfected cells treated for 6 hr with Veh, E_2_, or Gen. (*H*) Microarray profile showing comparison of Dex + E_2_ and Dex + Gen treatment, as sorted by Venn diagram. (*I*) Dex + E_2_ and Dex + Gen co-regulated genes separated by the direction of regulation; one representative gene for each pattern of regulation is displayed (induced, repressed, anti-correlated, and antagonized, from left to right). For all QRT‑PCR experiments, values were normalized to the housekeeping gene *PPIB*. Values shown are mean ± SE of four biological replicates except where indicated. *p < 0.05. **p < 0.01.

E_2_ regulates almost 3,000 genes in immortalized human uterine endometrial adenocarcinoma cells, and the spectrum of regulation largely overlaps with those genes regulated by glucocorticoids (Whirledge and Cidlowski 2013; Whirledge et al. 2013). Interestingly, E_2_ coadministered with Dex in these cells altered and antagonized glucocorticoid-induced gene expression over a range of concentrations (0.01–10 nM E_2_) (Whirledge and Cidlowski 2013). To determine whether environmental estrogens are also able to antagonize glucocorticoid-induced gene expression, we used QRT-PCR to quantify *GILZ* mRNA in Ishikawa cells after stimulation with Dex, E_2_, Gen, BPA, or Dex + E_2_. At a time point where Dex significantly up-regulated *GILZ* mRNA, E_2_ and Gen—but not BPA—antagonized the effect of Dex (Figure 1A). Antagonism by Gen was significant at 6 and 24 hr (Figure 1B). To determine the physiological relevance of Gen antagonism, we evaluated the range of concentrations at which Gen antagonized glucocorticoid-induced gene expression (*GILZ*) in cells treated with 1–1,000 nM Gen or 100 nM Dex (Figure 1C). At all Gen concentrations evaluated, Dex-induced *GILZ* gene expression was significantly repressed. At the concentration of 100 nM Gen, repression of glucocorticoid-induced *GILZ* expression was maximal; thus, we chose this concentration for all subsequent experiments.

Microarray analysis was performed following treatment with Gen or E_2_ to evaluate the transcriptional response to Gen in Ishikawa cells and determine the genome-wide common and unique targets of E_2_ and Gen. Surprisingly, less than one-third of the genes significantly regulated by Gen were in common with E_2_ treatment (268 of 932 gene probes) (Figure 1D). Most of the genes common to E_2_ and Gen treatment were regulated in a similar manner. However, some genes were identified as antagonistically regulated or anti-correlated, suggesting that E_2_ and Gen exposure may not result in the same transcriptional profile. Examples of the patterns identified are shown in Figure 1E. Furthermore, network analysis using IPA analysis indicated that genes regulated by Gen represent vastly different primary networks than those regulated by E_2_ (see Supplemental Material, Table S2). Gen has been reported to preferentially bind ERβ, compared with the affinity of E_2_ for ERα and ERβ (Manas et al. 2004). To evaluate whether preferential ligand binding is responsible for differences in gene expression profiles, we quantified mRNA of two genes with E_2_- and Gen-specific expression patterns following ERα or ERβ knockdown. Cells transfected with siRNA against ERα or ERβ, but not the non-targeting control pool, demonstrated significantly less ERα and ERβ mRNA, respectively (Figure 1F). Expression of angiotensinogen (*AGT*) and natriuretic peptide type C (*NPPC*) mRNA was evaluated in transfected cells following treatment for 6 hr with vehicle, E_2_, or Gen. *AGT* and *NPPC* were significantly induced by E_2_ and Gen (Figure 1G,H). Knockdown of ERα but not ERβ abolished E_2_ induction of *AGT* and *NPPC*, indicating that ERα likely mediates E_2_ regulation of these genes. Gen regulation of *AGT* also required ERα but not ERβ (Figure 1G). However, in the presence of *ER*α or *ER*β siRNA, Gen induction of *NPPC* was significantly less, suggesting Gen regulation of *NPPC* requires both ERα and ERβ.

Dex and E_2_ co-regulated global gene expression in Ishikawa cells (Whirledge et al. 2013). In light of the unique gene regulation by Gen, we compared the gene list of Dex + E_2_ with that of Dex + Gen. Interestingly, the combination of glucocorticoids and Gen regulated a set of genes unique from those regulated by glucocorticoids and E_2_ (Figure 1H). Though 1,016 genes were common to the Dex + E_2_ and Dex + Gen treatment groups, a significant number of these commonly regulated genes was anti-correlated or demonstrated antagonistic regulation when comparing the two treatment paradigms. In Figure 1I, we provide representative genes demonstrating all patterns of direction discovered. Furthermore, IPA analysis indicated that genes regulated by glucocorticoids and Gen represent distinct gene networks from those regulated by glucocorticoids and E_2_ (see Supplemental Material, Table S3). Those deviating gene networks indicate that Gen regulates a unique, and perhaps divergent, transcriptome in the presence of glucocorticoids in Ishikawa cells.

**Figure 2:**
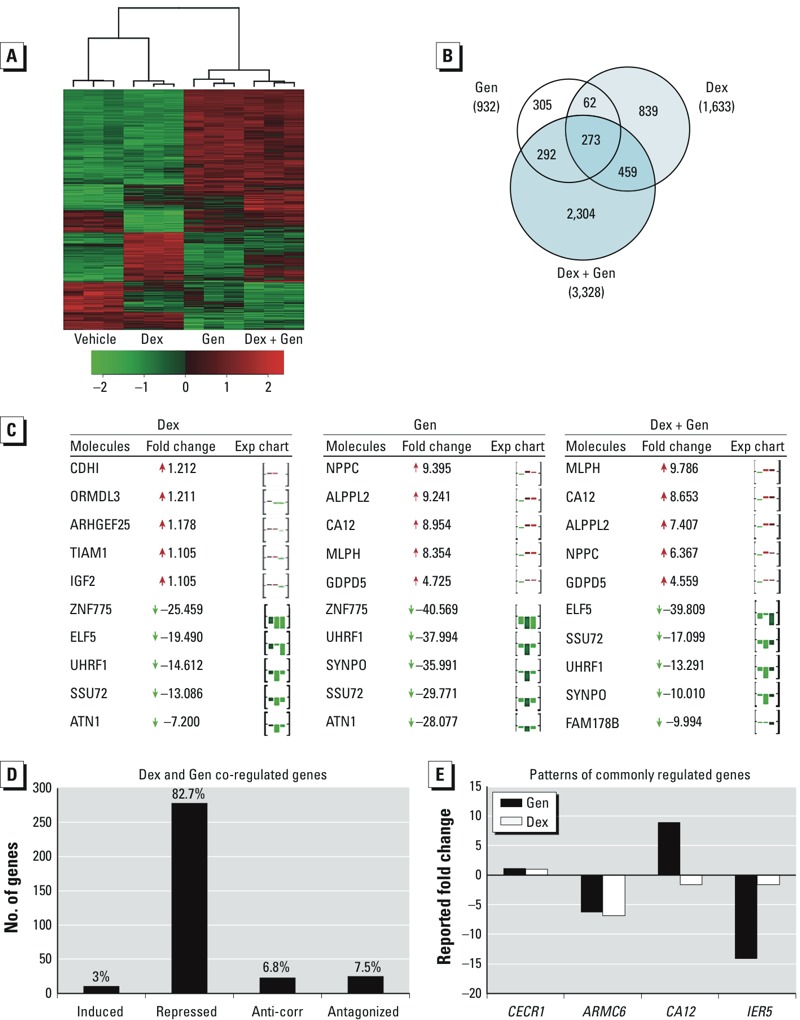
Microarray analysis revealed common and unique targets of Gen and Dex. (*A*) Number of probes regulated by Dex, Gen, and Dex + Gen from three biological replicates organized as either induced (red) or repressed (green) according to treatment group. (*B*) Venn diagram showing the number of probes that were statistically different (*p* < 0.01) between treatment groups. (*C*) The top five induced and repressed co-regulated by Dex, Gen, and Dex + Gen organized by treatment group; the expression (Exp) chart illustrates the relative fold change from vehicle for the Dex, Gen, and Dex + Gen treatment groups. (*D*) Dex, Gen, and Dex + Gen co-regulated genes were separated by direction of regulation. (*E*) One representative gene for each discovered pattern of regulation is displayed (induced, repressed, anti-correlated, and antagonized, from left to right).

Based on the unique transcriptional profile of Gen, microarray analysis was performed to identify genes regulated by both Dex and Gen in Ishikawa cells. Comparison of significantly regulated probes identified 5,893 genes regulated by Dex, Gen, or Dex + Gen. Gene profiles are shown as a heat map representing raw data (Figure 2A). Using Venn diagram analysis, we compared the gene lists to identify genes that are common and unique to each of the three treatment groups (Figure 2B). Gen treatment regulated 932 genes, Dex treatment regulated 1,633 genes; however, 3,328 genes were regulated only by the combination of Dex + Gen. Unexpectedly, two-thirds of the Dex + Gen genes were regulated only in the presence of Dex and Gen together and not by Dex or Gen alone, representing previously unidentified molecular gene targets.

Venn diagram analysis indicated that several genes were targets of both glucocorticoids and Gen. The top five induced and repressed genes by treatment are listed in Table 1. Several genes were regulated in all treatment groups, although expression levels differed by treatment. The expression chart for each gene illustrates the variation in regulation by treatment (Figure 2C). To examine the extent of transcriptional remodeling for Dex- and Gen-regulated genes, co-regulated genes from the overlapping gene lists were classified as *a*) induced by both Dex and Gen, *b*) repressed by both Dex and Gen, *c*) anti-correlated (genes with opposing direction of regulation), or *d*) antagonistically regulated (Figure 2D); a representative gene demonstrating each of these patterns is provided in Figure 2E. Almost 7% of commonly regulated genes (22 genes) were anti-correlated (see Supplemental Material, Table S4), and 25 genes were antagonistically regulated. Select genes representing different patterns of regulation were independently validated though the QRT-PCR analysis of four independent biological replicates not included in the initial microarray analysis (see Supplemental Material, Figure S1). Period circadian clock 1 (*PER1*) represented a Dex-induced gene and was validated as such. Leukemia inhibitory factor (*LIF*) was validated as a gene repressed in all treatment groups. *NPPC* and carbonic anhydrase 12 (*CA12*) were validated as Gen-induced genes, and interestingly, Dex antagonized Gen-induction for both genes. Guanosine monophosphate reductase (*GMPR*) was identified as a Dex-induced gene antagonized by Gen co-treatment and was validated as such. Microseminoprotein, beta (*MSMB*) demonstrated synergistic regulation, where the induction by Dex + Gen was greater than with Dex or Gen alone.

**Table 1:** Fold change of the top five induced and repressed genes by treatment.

Treatment, gene	Fold change
Dex
*PRR16*	11.71
*GMPR*	10.39
*PNMT*	9.46
*TDRD9*	8.84
*PER1*	8.60
*FRMD4A*	–9.23
*TMEM191B*	–15.05
*NEUROG1*	–16.06
*ELF5*	–19.49
*ZNF775*	–25.45
Gen
*CACNA1I*	20.67
*MSMB*	19.54
*NPPC*	9.39
*ALPPL2*	9.24
*CA12*	8.95
*FOXB1*	–23.44
*ATN1*	–28.07
*SYNPO*	–35.99
*GRIN1*	–37.20
*ZNF775*	–40.56
Dex + Gen
*MSMB*	30.26
*MICALCL*	16.38
*CACNA1I*	15.48
*CAMP*	12.98
*DHRS3*	11.49
*SP5*	–17.21
*NEUROG1*	–17.68
*TMEM191B*	–18.02
*ELF5*	–39.80
*ZNF775*	–45.33

**Figure 3:**
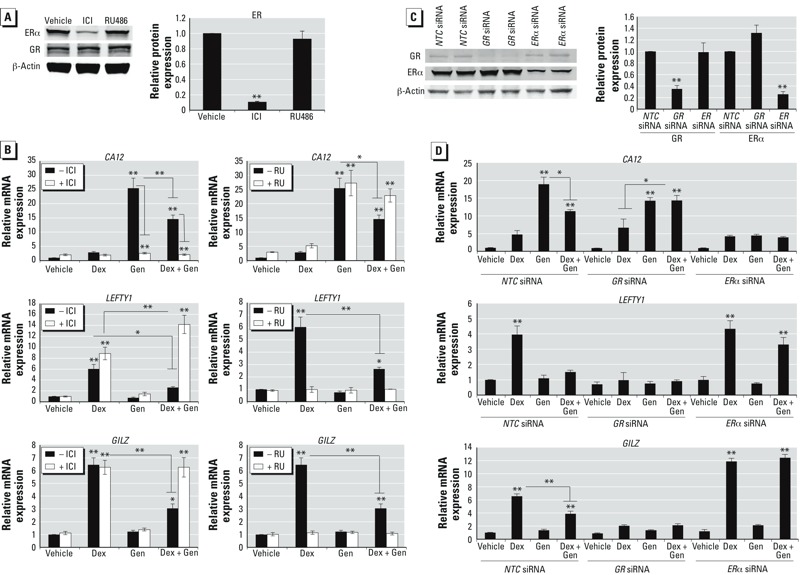
The GR and ER were required for transcriptional antagonism of three commonly regulated genes. (*A*) Cells treated 7 hr with the ER antagonist ICI or the GR antagonist RU486 assayed for ERα and GR protein levels by Western blot (left) and quantitated ER protein level (right). (*B*) mRNA expression of *CA12* (carbonic anhydrase 12; top), *LEFTY1 *(left-right determination factor 1; center), and *GILZ* (glucocorticoid-induced leucine zipper; bottom) in cells pretreated 1 hr with ICI (left) or RU486 (RU; right) and then treated with vehicle (Veh), Dex, Gen, or Dex + Gen for 6 hr. (*C*) GR and ERα protein levels in cells transfected with non-targeting control (NTC) siRNA, *GR* siRNA, or *ER*α siRNA as assessed by Western blotting (left), and the extent of knockdown compared with NTC (right). (*D*) mRNA expression of *CA12* (top), *LEFTY1* (center), and *GILZ* (right) in cells transfected with NTC siRNA, *GR* siRNA, or *ER*α siRNA and treated for 6 hr with vehicle, Dex, Gen, or Dex + Gen. All protein values were normalized to β-actin and compared with vehicle-treated cells; all mRNA values were normalized to the housekeeping gene *PPIB*. Values are presented as mean ± SE of four biological replicates. *p < 0.05. **p < 0.01.

To determine whether GR and ER are required for transcriptional regulation by Dex and Gen in common gene targets, cells were pretreated for 1 hr with either the GR antagonist RU486 or the ER antagonist ICI; gene expression of select co-regulated genes was analyzed 6 hr after treatment with Dex, Gen, or Dex + Gen (Figure 3). To confirm that ICI exposure, which competitively binds, down-regulates, and degrades both ERα and ERβ, resulted in lower ER expression, we performed Western blot analysis on whole cell lysates from cells treated for 7 hr with ICI or RU486 (Figure 3A). ICI significantly decreased ERα protein levels. RU486 did not significantly alter GR or ER protein levels (data not shown), but the absence of *GILZ* mRNA induction following Dex treatment indicated that GR-actions were blocked and the efficacy of this antagonist. In the microarray and by independent validation, *CA12* mRNA was induced by Gen and antagonistically regulated by Dex. Induction of *CA12* mRNA was blocked when cells were pretreated with the ER antagonist ICI (Figure 3B). Dex antagonism of Gen-mediated *CA12* induction in the Dex + Gen treated cells was fully relieved by pretreatment with the GR antagonist RU486, suggesting that both receptors are likely involved in co-regulation of *CA12*. Antagonistic regulation of left right determination factor 1 (*LEFTY1*) by Dex and E_2_ in Ishikawa cells was previously shown to be mediated by GR and ER (Whirledge et al. 2013). Similar to E_2_, Gen antagonized GR-mediated Dex-induced *LEFTY1* expression (Figure 3B). Gen antagonism was blocked by ICI, and RU486 blocked Dex-induction of *LEFTY1* mRNA expression, indicating that co-regulation of *LEFTY1* gene expression by glucocorticoids and Gen requires GR and ER. In human uterine endometrial adenocarcinoma cells, as well as in the mouse uterus, Dex and E_2_ have been reported to regulate the expression of the *GILZ* gene through GR and ERα (Whirledge and Cidlowski 2013). Dex-induced expression of *GILZ* was also antagonized by Gen treatment, and the ER antagonist ICI abrogated this antagonism (Figure 3B).

Analysis of gene expression in the presence of GR and ER inhibitors indicates that these receptors are required for the antagonistic regulation of *CA12*, *LEFTY1*, and *GILZ* by Dex and Gen. To evaluate the requirement of GR and ERα more specifically, cells were transfected with siRNA against *GR* and *ER*α prior to agonist treatment. Transfection with siRNA against *GR* and *ER*α, but not the non-targeting control pool, was able to significantly reduce the expression GR and ERα protein without affecting levels of the housekeeping gene β-actin (Figure 3C). Cells transfected with non-targeting control pool, *GR*, or *ER*α siRNA and treated for 6 hr with vehicle, Dex, Gen, or Dex + Gen were examined for *CA12*, *LEFTY1*, and *GILZ* mRNA expression by QRT-PCR (Figure 3D). Knockdown of GR expression by siRNA abrogated the ability of Dex to repress Gen-induced *CA12* mRNA expression. Furthermore, ERα knockdown eliminated induction of *CA12* by Gen, confirming the necessity of GR and ERα in the co-regulation of *CA12*. For both of the glucocorticoid-induced genes, repression of GR by siRNA attenuated *LEFTY1* and *GILZ* induction by Dex. ERα knockdown by siRNA relieved the antagonism brought about by Gen treatment. Interestingly, induction of *GILZ* mRNA by Dex increased in cells transfected with *ER*α siRNA.

Based on the mechanism discovered for the co-regulation of *GILZ* by glucocorticoids and E_2_, transcriptional regulation of *GILZ* was more closely examined to determine whether Gen and E_2_ share a common mechanism of antagonistic regulation. To understand the mechanism by which Gen antagonized glucocorticoid-induced *GILZ* expression, treated cells were evaluated for evidence of indirect or direct regulation by Gen. Cells were administered cycloheximide 1 hr prior to hormone treatment, and *GILZ* mRNA levels were quantified 6 hr after treatment (see Supplemental Material, Figure S2A). Cycloheximide pretreatment did not alter the ability of Gen to repress Dex-induced *GILZ* mRNA expression. Nascent *GILZ* RNA expression in response to Dex and Dex + Gen treatment was examined to determine whether Gen antagonism of glucocorticoid-induced gene expression is a function of impeding transcriptional initiation (see Supplemental Material, Figure S2B). Co-treatment with Dex and Gen represses mature and nascent RNA transcripts. These data indicate Gen directly regulated glucocorticoid-induced *GILZ* expression at the level of transcription. Analysis of GILZ protein expression following 24-hr treatment indicates that changes in mRNA message levels are translated into differences in protein expression (see Supplemental Material, Figure S2C).

**Figure 4:**
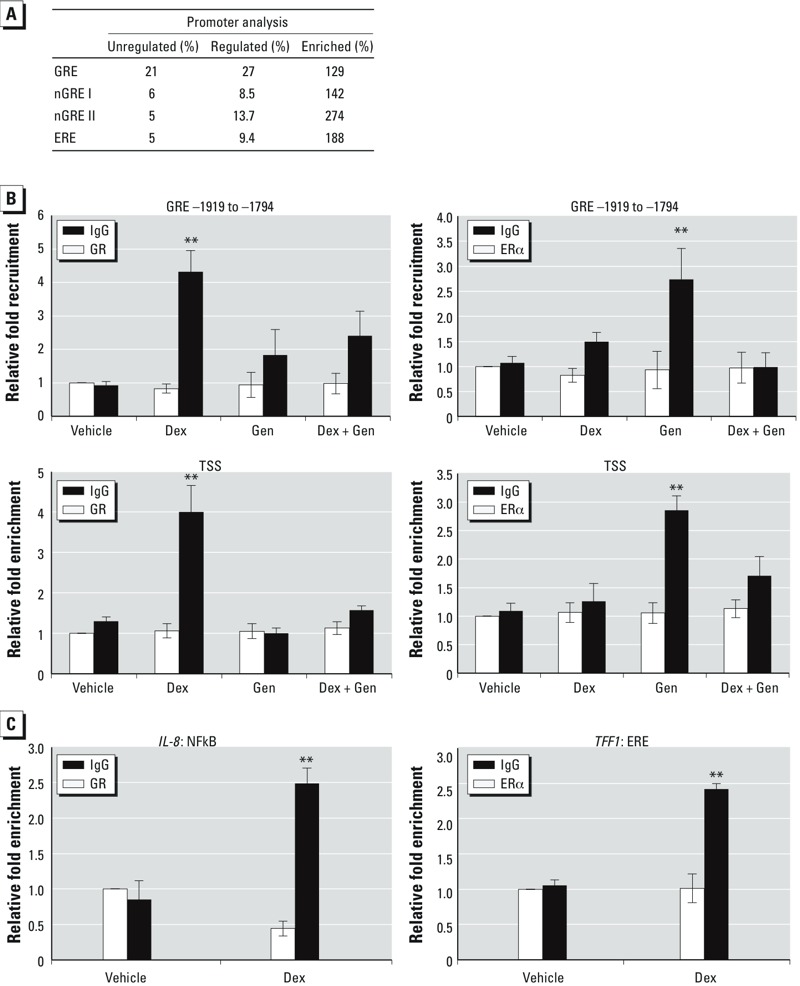
Enhanced promoter recruitment represents one mechanism of glucocorticoid and Gen transcriptional regulation. (*A*) The JASPAR CORE Vertebrata server was used to search a 3,500‑bp region around the transcriptional start site of annotated genes, and antagonistically regulated genes were compared with 100 genes from the microarray platform that were not significantly regulated by Dex or Gen. (*B*) Recruitment of GR (left) and ERα (right) to the GRE at position –1919 to –1794 (top) and to the transcriptional start site (TSS; bottom) assessed by ChIP assay in cells treated with vehicle, Dex, Gen, or Dex + Gen for 1 hr; enrichment of the sequences containing the GRE and TSS promoter region was measured by QRT‑PCR. (*C*) Recruitment of GR to an NFκB site in *IL-8* (left) and ERα to an ERE in *TFF1* (right) analyzed by ChIP and quantified by QRT‑PCR; all values were normalized to input and set relative to vehicle IgG. Values shown are mean ± SE of at least five biological replicates. **p < 0.01.

A narrow *in silico* promoter analysis was performed on genes identified as anti-correlated or antagonistically regulated to identify response elements for GR and ER. The enrichment of GREs and estrogen response elements (EREs) would suggest that one mechanism by which these genes are regulated by glucocorticoids and Gen is through their respective receptors binding promoter elements. The open-access JASPAR database of matrix-based transcription factor binding profiles (Bryne et al. 2008) was used to search 3,000 bp upstream and 500 bp downstream of the TSS of each gene. We determined that 319 genes were anti-correlated or antagonistically regulated and that 233 genes contained an annotated promoter. To determine enrichment of response elements, genes with an annotated promoter were analyzed for the inclusion of a GRE, nGRE (sequence I and II), or ERE sequence and compared with 100 genes found in the microarray to not be significantly regulated by Dex or Gen (Figure 4A). Compared with unregulated genes, antagonized genes demonstrated an enrichment of 129% for GREs, 142% for nGRE I, 274% for nGRE II, and 188% for the ERE sequence. The enhanced presence of GR and ER response elements in co-regulated genes may signify the ability of these steroid receptors to directly regulate the expression of target genes.

Given that ERα was required for the Gen-mediated antagonism of GR-induced *GILZ* expression, ChIP assays were performed on human genomic DNA from Ishikawa cells treated for 1 hr with vehicle, Dex, Gen, or Dex + Gen to examine the recruitment of ERα to the *GILZ* promoter *in vitro* (Figure 4B). Occupancy of GR and ERα at the GRE located at position –1919 to –1794 in the promoter and the TSS of *GILZ* following Dex and E_2_ stimulation in human endometrial cells was described previously (Whirledge and Cidlowski 2013). In a mechanism similar to the E_2_-like antagonism of Dex-induced *GILZ* gene expression, both GR and ERα were recruited to the GRE located at –1919 to –1794 and the transcriptional start site of *GILZ* in the presence of Dex or Gen, respectively (Figure 4B). In the presence of Dex + Gen, GR association with the chromatin at the GRE –1919 to –1794 and the transcriptional start site were reduced, offering a potential mechanism by which glucocorticoid-mediated up-regulation of *GILZ* mRNA was antagonized by the presence of Gen and glucocorticoids. These data suggest that E_2_ shares both common and divergent mechanisms of gene regulation and interactions with other transcription factors. Recruitment of GR to a nuclear-factor kappa B (NFκB) site in interleukin 8 (*IL-8*) and ERα to an ERE in trefoil factor 1 (*TFF1*) served as controls (Figure 4C). One hour after treatment with the same concentrations of Dex that induced recruitment of GR to the promoter of GILZ, the occupancy of GR near a NFκB site in *IL-8* was significantly enhanced compared with vehicle treatment. ERα occupancy of the evaluated ERE in the *TFF1* promoter was significantly increased at the same concentration of Gen used to induce ERα recruitment to the GILZ promoter following 1 hr treatment.

## Discussion

Reported pathologies of the female reproductive tract in animal models that have been attributed to Gen exposure have raised concerns regarding the widespread inclusion of soy in commercially processed food and as a large constituent of the infant formula market (Barrett 2006). The present study provides important evidence that Gen exposure induces a significantly different transcriptional response than E_2_ in Ishikawa cells. We previously showed that the endogenous estrogen E_2_ regulated GR-mediated transcription in Ishikawa cells *in vitro* and in the mouse uterus (Whirledge and Cidlowski 2013; Whirledge et al. 2013). In the present study, we observed through whole genome microarray analysis that the response to Gen alone and in combination with Dex was significantly different from that of E_2_ or Dex + E_2_ under the same conditions. In fact, most of the gene probes regulated by Gen were not in common with E_2_. Gen-regulated genes represented distinct gene networks, suggesting that Gen regulates distinct biological pathways in these immortalized endometrial cells. Whether this represents the actual physiology of normal endometrium will require additional studies in mouse and human cell model systems. In the presence of Dex, Gen regulated unique genes independently of E_2_. These differences may reflect altered ERα and ERβ utilization by Gen, compared with E_2_, or Gen regulation of expression of other steroid receptors. Gen also demonstrates potent tyrosine kinase inhibitor activities (Akiyama et al. 1987). However, these effects occur at much higher concentrations than those used in this study (10–100 μM); when the results of the receptor knockdown experiments are considered, the glucocorticoid antagonist properties of Gen appear to be mediated through ERα for those genes studied (Barnes et al. 2000).

The interactions between steroid receptors are poorly understood, including the mechanisms by which GR and ERα regulate gene transcription. Altered GR and ERα recruitment to the promoter of the glucocorticoid-induced gene *GILZ* mediates the antagonistic regulation by Dex and E_2_ (Whirledge and Cidlowski 2013). However, the mechanism of co-regulation by GR and ER has not yet been discovered for those newly identified common targets of Dex and Gen. Evaluation of global transcription factor binding in breast cancer cells has indicated that GR and ER can mediate genomic cross-talk by regulating each other’s binding at recognition sites; this action allows rapid reprogramming of the chromatin structure and targeting of novel genes following the co-activation of both receptors (Miranda et al. 2013). This model of transcription factor interplay is likely responsible for the regulation of those common gene targets, as well as genes found only to be induced or repressed in the presence of Dex + Gen. Interestingly, less than one-third of genes co-regulated by Dex + Gen are the same genes regulated by Dex + E_2_. Thus, in the model of molecular interplay between GR and ER, the unique pattern of co-regulation by Dex + Gen may indicate that Gen-bound ER is recruited to different recognition sites or results in alternative cofactor recruitment compared with ER bound to the endogenous ligand (Chang et al. 2008). In fact, altered genome-wide ERα binding following Gen treatment is evident in endometrial cancer cells and suggests one mechanism by which E_2_ and Gen treatments result in different patterns of gene expression (Gertz et al. 2012).

To understand how glucocorticoids and Gen regulate common genes, we evaluated three novel targets in greater detail. Our study is the first to report transcriptional regulation of *CA12*, encoding a membrane-associated protein responsible for the acidification of the microenvironment, by Gen in an immortalized human uterine cell line. In the female reproductive tract, CA12 is localized to the endometrium in both the mouse and human uterus, where maintenance of appropriate pH levels is critical to the fertilization process (Hynninen et al. 2004; Karhumaa et al. 2000). CA12 is found at high levels in the proliferative endometrium, a phase characterized by high levels of E_2_, suggesting that aberrant induction by environmental estrogens may disrupt the precision of timing necessary for fertilization (Ivanov et al. 2001). Although one previous report indicated that CA12 is a target of GR signaling (Endröczi et al. 1994), glucocorticoids are able to accelerate the enzymatic activity of carbonic anhydrases (Donn et al. 2007). The ability of endogenous cortisol (which varies with the menstrual cycle) to antagonize Gen-induced *CA12* gene expression is unknown. Similarly, the degree to which aberrant CA12 expression compromises fertility in not understood (Nepomnaschy et al. 2011).

Transcriptional regulation of *LEFTY1* and *GILZ* by Dex and the environmental estrogen Gen has not been previously reported (Whirledge and Cidlowski 2013; Whirledge et al. 2013). The *in vivo* significance of glucocorticoid regulation of *LEFTY1* and *GILZ* in the uterus is not clear, but both LEFTY1 and GILZ regulate important biological functions. LEFTY1 has been shown to play an important role in uterine decidualization and embryo implantation (Tabibzadeh 2011). Furthermore, LEFTY1 is temporally expressed in the endometrium of fertile women (Kothapalli et al. 1997). Alterations to the precise timing of GILZ expression may also adversely affect uterine biology, especially the immunomodulatory effects of GILZ. GILZ is an important mediator of activation, function, and cell death of T lymphocytes, immune cells essential to implantation and early stages of pregnancy (Ayroldi and Riccardi 2009; Nevers et al. 2011). E_2_ and Gen share a similar mechanism by which glucocorticoid-induced *GILZ* expression is antagonized. However, dietary Gen exposure does not follow the natural patterns of cyclic E_2_ and could result in aberrant GILZ expression. The consequences of this are unknown but have the potential to directly affect immune tolerance within the uterus.

## Conclusion

Although Gen has been found to be clinically beneficial in relation to cardiovascular disease and cancer (Adlercreutz et al. 1992; Beaglehole 1990), research indicates that consuming environmentally relevant doses of Gen has adverse effects on the female reproductive tract in mammals (Bennetts et al. 1946; Setchell et al. 1987). Modulation of ER activity is partially responsible for the uterotrophic effects of Gen, and accordingly, we observed some overlap between Gen- and E_2_-regulated genes in Ishikawa cells; however, two-thirds of Gen-regulated genes were unique to this treatment. Furthermore, in the presence of Gen, Dex exposure resulted in a divergent pattern of gene regulation. Our findings demonstrate one mechanism by which Gen may directly regulate GR-mediated gene expression and represent an important *in vitro* model to discover the molecular actions of Gen. In addition to the select genes studied, co-regulated genes identified by genomic analysis may provide exciting molecular targets with potential biological insight.

## Supplemental Material

(515 KB) PDF
