# Supplementary material for: Genistein Disrupts Glucocorticoid Receptor Signaling in Human Uterine Endometrial Ishikawa Cells
Source: Environ Health Perspect. 2014 Aug 19;123(1):80–7. doi: 10.1289/ehp.1408437 (PMC4286279; doi:10.1289/ehp.1408437)
Supplement: (515 KB) PDF [file ehp.1408437.s001.508.pdf]

Environmental Health Perspectives doi:10.1289/ehp.1408437

Supplemental Material

Genistein Disrupts Glucocorticoid Receptor Signaling in Human Uterine Endometrial Ishikawa Cells

Shannon Whirledge, Linda T. Senbanjo, and John A. Cidlowski

RNA isolation and QRT-PCR

Total RNA was extracted from Ishikawa cells using the QIAGEN QIAshredder and RNeasy mini kit (Qiagen, Valencia, CA) according to the manufacturer's protocol. Deoxyribonuclease (DNase) treatment was performed on-column using a ribonuclease-free DNase Kit (Qiagen) according to the manufacturer's instruction. Samples were quantified by Nanodrop ND-1000 (Thermo Scientific, Wilmington, DE) spectrophotometer and purity was analyzed by the 260:280 absorbance ratio. Quantitative real-time reverse transcription-PCR (QRT-PCR) was performed on isolated RNA by using the 7900HT sequence detection system and predesigned primer/probe sets from Applied Biosystems (Foster City, CA) according to the manufacturer's instructions. QRT-PCRs were performed on 100 ng total RNA using the One-Step QRT-PCR Universal Master Mix reagent. Standard curves were generated by serial dilution of isolated total RNA.

Microarray study

Starting with 500 ng of total RNA, Cy3 labeled cRNA was produced according to manufacturer's protocol. For each sample, 1.65ug of Cy3 labeled cRNAs were fragmented and hybridized for 17 hr in a rotating hybridization oven. Slides were washed and then scanned with an Agilent Scanner. Data was obtained using the Agilent Feature Extraction software (v9.5), using the 1-color defaults for all parameters. The Agilent Feature Extraction Software performed error modeling, adjusting for additive and multiplicative noise. The resulting data were processed using the Rosetta Resolver® system (version 7.2) (Rosetta Biosoftware, Kirkland, WA). Principal Component analysis was performed on all samples and all probes to reduce the dimensionality of the data while preserving the variation in the data set. This allowed assessment of the similarities and differences between samples within a treatment group and between treatment groups. Error-weighted ratios that Resolver generates and the associated p value were

used to identify differentially expressed probes. Gene lists were generated from the average of three biological replicates from each hormone treatment by selecting probes that were significant at $p < 0.01$ as determined by ANOVA.

Whole cell lysates

Cells grown *in vitro* were washed twice with ice-cold PBS, treated with 350 μl radioimmunoprecipitation buffer with the addition of protease inhibitor cocktail tablets (cOmplete Mini, EDTA-free, Roche, Indianapolis, IN), scraped off the plate, and rotated at $4^{o}C$ for 30 min. Cellular debris was removed by centrifugation at 13,200 rpm (16,100 relative centrifugal force) for 20 min in a tabletop Eppendorf 5415R Centrifuge (Eppendorf International, Hauppauge, NY) at $4^{o}C$ and supernatant collected. Protein concentration was determined using a BCA protein quantitation kit (Pierce, Rockford, IL). Sample buffer (5X) was added to samples containing 40 μg of protein, heated to $95^{o}C$ for 5 min, and separated on a 10% ReadyGel Tris-Gly gels (Bio-Rad, Hercules, CA). Proteins were then transferred onto a nitrocellulose membrane. Membranes were blocked for 1 hr with 10% skim milk in TBST (Tris-buffered saline with 0.1% Tween 20). Immunoreactivity was visualized by incubation with a mixture of goat anti-rabbit Alexa® Fluorophore 680 conjugated (Molecular Probes, Carlsbad, CA) and goat anti-mouse IRDye 800 conjugated secondary (Rockland Immunochemicals, Gilbertsville, PA) antibodies for 1 hr at room temperature and scanned on the Odyssey LiCor imaging system.

Chromatin immunoprecipitation assay

Briefly, cells were plated in 150-mm dishes in RPMI Medium 1640. After 24 hr, growth media was replaced with phenol red-free RPMI 1640 containing 5% charcoal dextran treated (stripped) FBS. The cells were grown for an additional 24 hr before treatment with either vehicle, 100 nM

Dex, 100 nM Gen, or 100 nM Dex and 100 nM Gen for 1 hr. The cells were fixed in 1% formaldehyde and harvested in lysis buffer containing protease inhibitors (cOmplete Mini, EDTA-free, Roche, Indianapolis, IN). The nuclear contents were then sonicated using a Branson Sonifier 150 at setting 4 with 10-sec pulses, four times on ice. Sheared chromatin was precleared with rabbit IgG and protein A agarose/salmon sperm DNA. After elution of protein:DNA complexes and DNA purification, PCR analysis was performed on immunoprecipitated and input DNA.

Figure S1

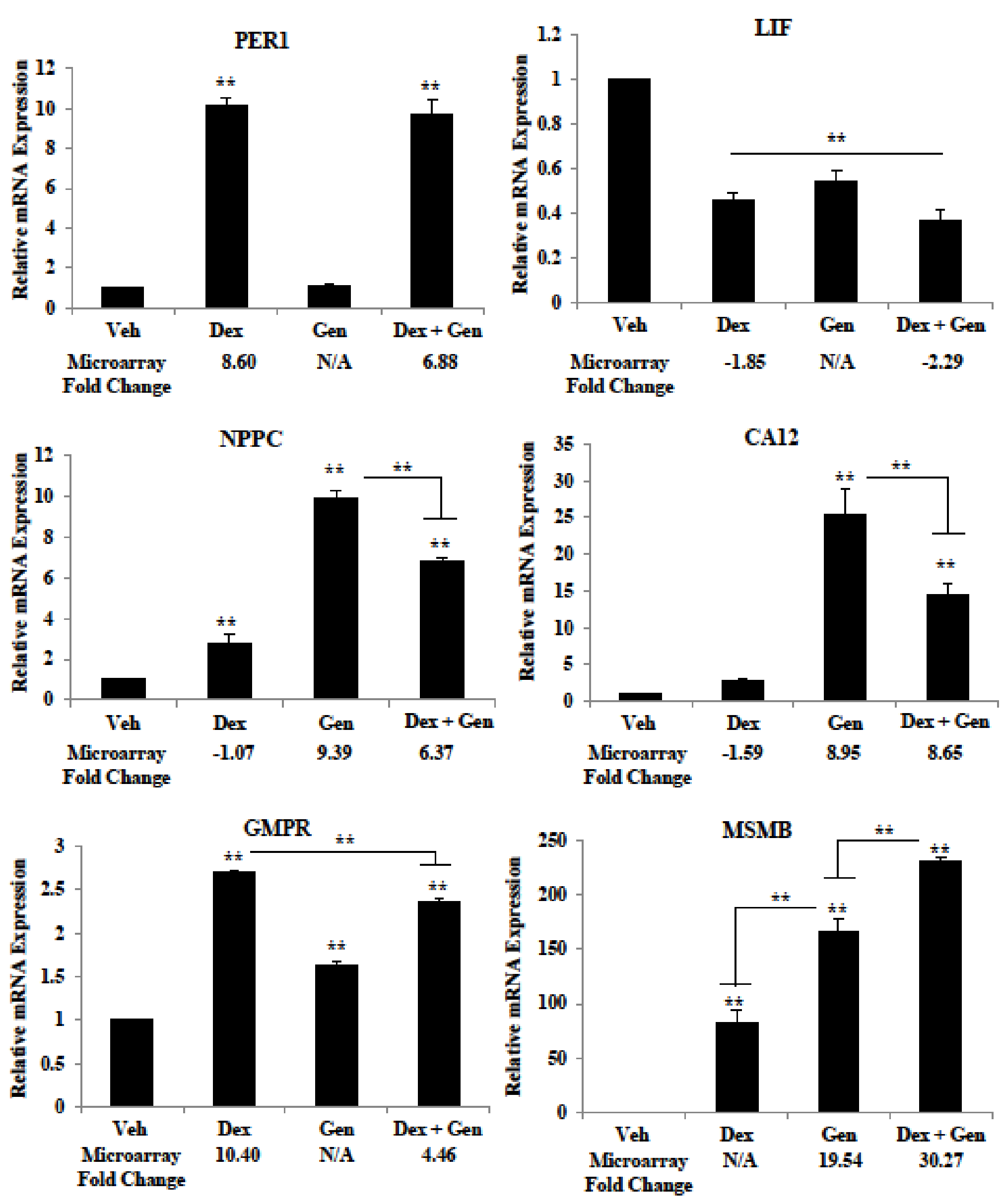

Figure S1. Independent Validation of Dex and Gen Co-Regulated Genes. Ishikawa cells were administered vehicle, 100 nM Dex, 100 nM Gen, or 100 nM Dex and 100 nM Gen, and mRNA was evaluated by quantitative RT-PCR. Gene expression was determined for Period Homolog 1 (*PER1*), Leukemia Inhibitory Factor (*LIF*), Natriuretic Peptide Type C (*NPPC*), Carbonic

Anhydrase 12 (*CA12*), Guanosine Monophosphate Reductase (*GMPR*), and Microseminoprotein, Beta (*MSMB*). Values were normalized to the housekeeping gene Cyclophilin B (*PPIB*). Bar graphs show mean ± SEM of four biological replicates. (** $p < 0.01$) The fold change valued determined by microarray for each gene is listed below each treatment. N/A indicates that the gene was not found to be significantly regulated by treatment in the microarray.

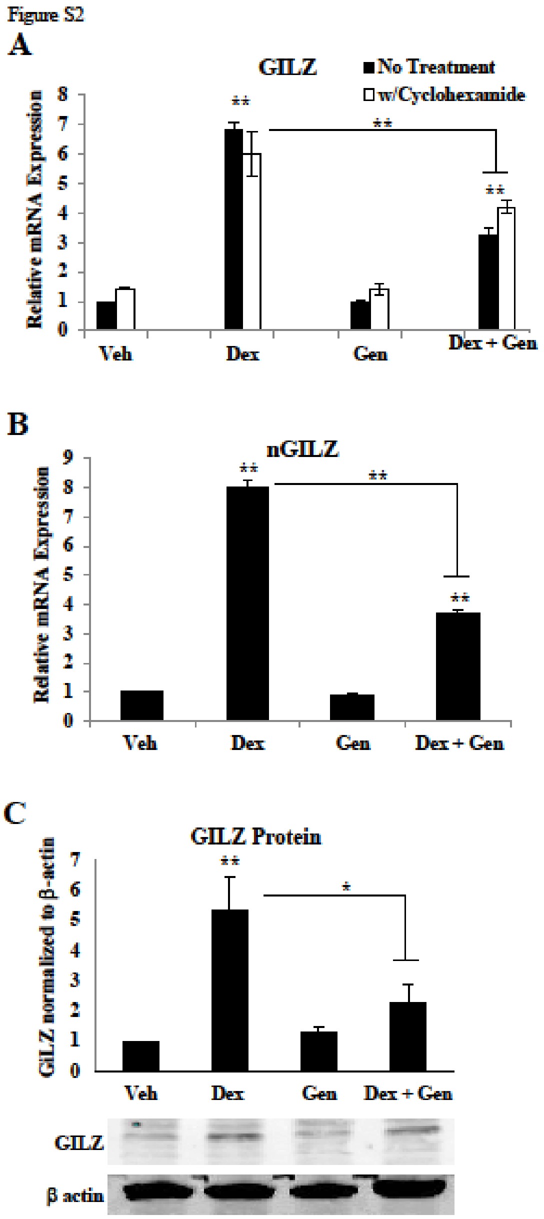

Figure S2. Antagonistic regulation of the Glucocorticoid-induced Leucine Zipper (*GILZ*) gene by dexamethasone and genistein is direct. A) Cells were treated +/- with 10 μM cycloheximide for 1 hr prior to agonist treatment and then treated with 100 nM Dex, 100 nM Gen, or 100 nM Dex and 100 nM Gen. mRNA evaluated by QRT-PCR for Glucocorticoid-Induced Leucine

Zipper (*GILZ*). (n=4; ** $p < 0.01$) B) Relative levels of nascent RNA was measured by QRT-PCR in cells following treatment with vehicle, Dex, Gen, or Dex and Gen for 6 h. Values were normalized to nascent RNA for the housekeeping gene Cyclophilin B (*PPIB*). (n=4; ** $p < 0.01$) C) Cells were treated for 24 hr with Dex, Gen, or Dex and Gen and assayed for GILZ protein by Western blot. GILZ expression levels were normalized to protein levels of the housekeeping gene β-actin. (n=4; ** $p < 0.01$)

Table S1. Chromatin Immunoprecipitation Primers.

Locus	Primer 1	Primer 2
GRE 1919-1794	5'-GCTCAGAGACTTTGTGCGTATTTGG-3'	5'-AGGCTTGATCAGAGAGGTTTG-3'
TSS	5'-AAAGCCCGGTACAGGACTCCATTTG-3'	5'-ACCTCGTATGTCACAAACTCCACG-3'
IL-8 NFkB	5'-AGTGTGATGACTCAGGTTTGCCCT-3'	5'-TCCTAGAAGCTTGTGTGCTCTGCT-3'
TFF1 ERE	5'-TGGGCTTCATGAGCTCCTTCCCTT-3'	5'-GATTCATAGTGAGAGATGGCCGGA-3'

Table S2. Top Regulated Networks of Estradiol and Genistein Genes.

Network	Score[a]	Focus molecules
**Estradiol**		
1. Connective Tissue Development and Function, Embryonic Development, Organ Development	37	33
2. Lipid Metabolism, Small Molecule Biochemistry, Cell Morphology	35	32
3. Developmental Disorder, Hereditary Disorder, Neurological Disease	35	32
4. Endocrine System Development and Function, Small Molecule Biochemistry, Tissue Morphology	35	32
5. Cellular Development, Embryonic Development, Organismal Development	32	32
**Genistein**		
1. Embryonic Development, Organismal Development, Tissue Development	39	28
2. Tissue Morphology, Protein Synthesis, Cell Death and Survival	33	25
3. Connective Tissue Development and Function, Embryonic Development, Organ Development	31	25
4. Connective Tissue Development and Function, Embryonic Development, Organ Development	31	24
5. Cellular Assembly and Organization, Cellular Function and Maintenance, Cellular Movement	31	24

[a]The score is a measure of the number of focus genes in a network.

Table S3. Top Regulated Networks of Dex + E_2 and Dex + Gen Unique Genes.

Network	Score[a]	Focus molecules
**Dex + E_2 Unique**		
1. Cellular Movement, Reproductive System Development and Function, Connective Tissue Disorders	34	35
2. Lipid Metabolism, Small Molecule Biochemistry, Cellular Compromise	31	34
3. Embryonic Development, Organismal Development, Cellular Movement	31	34
4. Cellular Assembly and Organization, Cell Cycle, DNA Replication, Recombination and Repair	31	34
5. Protein Degradation, Protein Synthesis, Immunological Disease	31	34
**Dex + Gen Unique**		
1. Cell-To-Cell Signaling and Interaction, Inflammatory Response, Neurological Disease	38	34
2. Gene Expression, Cell Death and Survival, Cellular Development	33	32
3. Renal and Urological Disease, Cellular Development, Cardiovascular Disease	33	33
4. Molecular Transport, Cell-To-Cell Signaling and Interaction, Cellular Assembly and Organization	31	31
5. Cellular Assembly and Organization, Hair and Skin Development and Function, Developmental Disorder	30	31

[a]The score is a measure of the number of focus genes in a network.

Table S4. Dex and Gen Anti-Correlated Genes.

Sequence name	Sequence description	Gen (fold change)	Dex (fold change)	Dex + Gen (fold change)
HERC2	hect domain and RLD 2	-1.1099	1.01872	
PPARGC1B	peroxisome proliferator-activated receptor gamma, coactivator 1 beta	1.38709	-1.11786	
THC1953783	Unknown	1.02979	-1.61357	
NPPC	natriuretic peptide C	9.39464	-1.06704	6.36735
ALPPL2	alkaline phosphatase, placental-like 2	9.24051	-1.77678	7.40709
CA12	carbonic anhydrase XII	8.95392	-1.6438	7.7799
MLPH	melanophilin	8.35403	-1.3155	9.7861
GDPD5	glycerophosphodiester phosphodiesterase domain containing 5	4.72484	-1.01513	4.55899
ZNF418	zinc finger protein 418	1.06322	-1.0222	-1.29401
MGC4473	hypothetical LOC79100	1.00529	-1.08695	-1.45482
ENST00000333740	Unknown	-1.00175	1.06777	-1.22608
C9orf166	Transcribed locus	-1.00272	1.07483	-1.63403
SPDYE1	speedy homolog E1 (Xenopus laevis)	-1.00989	1.06938	-1.87445
ZBTB47	zinc finger and BTB domain containing 47	-1.05266	1.01254	-1.38419
SHARPIN	SHANK-associated RH domain interactor	-1.09572	1.00316	-1.29075
A_24_P584936	Unknown	-1.09843	1.05868	-1.88452
BBX	bobby sox homolog (Drosophila)	-1.15876	1.003	-1.35218
C17orf54	chromosome 17 open reading frame 54	-1.16551	1.04161	-1.700922
ORMDL3	ORM1-like 3 (S. cerevisiae)	-1.17778	1.21066	-1.55611
NFE2L1	nuclear factor (erythroid-derived 2)-like 1	-1.20257	1.10098	-1.35131
MEIS3	Meis homeobox 3	-1.35095	1.03061	-1.58307
THC1909811	LMB1_HUMAN Laminin beta-1 chain precursor (Laminin B1 chain). [Human] {Homo sapiens}, partial (4%) [THC1909811]	-1.48693	1.07275	-1.44048
